# How Do Motor and Sensory Function Correlate with Daily Performance Recovery after Post-Stroke Robotic Intervention? A Secondary Analysis of a Non-Randomized Controlled Trial

**DOI:** 10.3390/biomedicines11030853

**Published:** 2023-03-10

**Authors:** Mª Pilar Rodríguez-Pérez, Patricia Sánchez-Herrera-Baeza, Rebeca Montes-Montes, Roberto Cano-de-la-Cuerda, Rosa M. Martínez-Piédrola, Sergio Serrada-Tejeda, Paula Obeso-Benítez, Marta Pérez-de-Heredia-Torres

**Affiliations:** Department of Physical Therapy, Occupational Therapy, Rehabilitation and Physical Medicine, King Juan Carlos University, Avenida de Atenas s/n, 28922 Alcorcón, Spain

**Keywords:** focal vibration, functionality, hand, rehabilitation, robotic, stroke, upper limb, vibration

## Abstract

New technologies have been developed to complement conventional interventions to better target the specific needs of people with stroke, and they have been shown to improve both function and performance. However, it is unknown whether the baseline levels of sensorimotor function and performance interrelate with the improvement in upper limb and daily performance. Thus, the aim of this study was to examine the relationship between baseline levels of sensorimotor function and daily performance and its impact on post-intervention improvement in people with stroke following a robotic intervention. A single-blind, non-randomized, controlled clinical trial was conducted. Participants in the experimental group (n = 9) received a robotic intervention in addition to conventional treatment. Sensorimotor function was measured with Semmes-Weinstein Monofilaments^®^ and the Fugl-Meyer Assessment Upper Extremity Scale. Upper limb and daily performance were measured with the MAL and SIS-16 scales. The multivariate regression models showed that baseline levels of upper limb performance and motor function predicted >95% of the variance in upper limb performance (*p* < 0.001), while pre-intervention levels of daily performance explained >75% of the post-intervention variance (*p* < 0.05). These findings indicate that basal upper limb motor function is associated with improved performance following a combined intervention of conventional treatment and robotic intervention.

## 1. Introduction

The World Health Organization defines stroke as “an acute focal neurological dysfunction caused by focal infarction at single or multiple sites of the brain” [1]. It is estimated that around 15 million people per year have a first-ever stroke, of whom 5 million experience subsequent permanent restrictions in their daily lives [2]. In this regard, a recent systematic review estimates that the cumulative incidence rate (CIR) worldwide is 11 per 100,000 inhabitants, and specifically, the CIR in European countries stands at 7, with men over 60 years of age being the profile with the highest prevalence of stroke in Spain. Despite the efforts made to reduce the occurrence of this pathology, stroke and its consequences continue to be major health and disability problems in the world population [3].

Stroke has important consequences on the functionality of individuals. In this respect, the International Classification of Functioning, Disability and Health (ICF) of the World Health Organization [4] provides a common theoretical framework for all health professions to operationalize aspects and factors related to health and daily functioning. The ICF classifies functioning and disability into (1) body functions and structures (physiological functions and anatomical parts of body systems) and (2) activities and participation (performance of a task or action; involvement in a life situation). The latter component can be further qualified by the domains of ability and performance, with the former being differentiated as the “performance of tasks in a uniform environment” (e.g., a clinical assessment of motor ability) and the latter as the “performance of tasks in the person’s actual environment” (e.g., an assessment of the person’s actual performance in daily activities and contexts). In the development of functionality and disability, the ICF considers two types of contextual factors: environmental factors and personal factors. Thus, over the last twenty years, the ICF has served as a reference for studying activity limitations and participation restrictions as consequences of impairments at the body function and structure levels. Furthermore, improving performance and participation in daily occupations is considered a main goal of stroke rehabilitation [5].

Several international reviews and both cross-sectional and longitudinal studies highlight the decline in activity performance and participation of people who have suffered a stroke [6,7,8,9,10,11]. The scientific literature indicates that contextual factors, including socioeconomic environmental factors and personal factors, such as age and gender, are significant predictors of functional recovery in this population [12,13]. Furthermore, functional recovery appears to be closely related to the previous level of performance and participation [9,11,14] and to the extent and severity of deficits in motor, sensory, and cognitive functions [6,8,14]. Therefore, the interdisciplinary intervention proposals aim at improving function, performance, and participation. In this regard, the incorporation of new technologies to conventional rehabilitation approaches allows for the systematization, specification, and operationalization of more tailored therapeutic elements, such as the intensity and repetition of certain tasks [15,16,17].

One of the main robotic systems used in the rehabilitation context as an adjuvant technique in the recovery of function and ability in people with stroke is the Amadeo^®^ manual robotic system [18]. This robot uses a fingertip sensor system that, through vibratory stimuli at different frequencies, provides proprioceptive input to the upper limb. Previous preliminary studies have explored the effectiveness of the Amadeo^®^ system for the recovery of motor functions after stroke [19,20]. Recently, a clinical trial conducted by our research group showed that the use of this system, in combination with conventional intervention, produces partial improvements in sensory and motor function in daily upper limb performance in terms of quantity and quality of movement, and in the overall performance of basic and instrumental activities of daily living [21]. However, it is still unknown whether baseline levels of sensory and motor function, as well as previous performance levels, are related to improvements in upper limb and daily activity performance following this intervention.

### Novelty of This Work

It has been reported that previous levels of function and performance may predict a better rehabilitation outcome in stroke patients. Furthermore, recent evidence suggests that multisensory stimulation delivered by robotic systems may be effective in improving sensory and motor function and even neuroplasticity in stroke patients. However, most research has explored the effectiveness in terms of gait function and performance [22], or in terms of upper limb motor function [23,24], and only few studies have focused on the effectiveness of robot systems in terms of both sensory and motor function and upper limb or overall performance [25]. While findings of our previous work suggest that a combination of conventional treatment and a robot system intervention may lead to partial improvements in sensorimotor function and both upper limb and overall daily performance [21], the relationship between baseline levels of function and performance and post-intervention improvements is still unclear. Exploring this relationship would contribute to a better prognosis and understanding of the specific variables predicting post-intervention outcomes following a stroke, and it would allow for a more tailored approach.

Therefore, the aim of this study is to perform a secondary analysis of the data from the clinical trial conducted by Rodriguez-Perez et al. [21] to determine the relationship between baseline levels of sensory and motor function and daily motor performance and their impact on the post-intervention improvement of performance in people with subacute and chronic stroke.

## 2. Materials and Methods

### 2.1. Design and Ethical Aspects

A single-blind, non-randomized, controlled clinical trial was conducted according to the Transparent Reporting of Evaluations with Non-Randomized Designs (TREND) guidelines. The study was approved by the Research Ethics Committee of the Universidad Rey Juan Carlos (code: 505202012620), and informed consent was obtained from all study participants. The study protocol was previously published in detail [21].

### 2.2. Participants and Group Assignments

Participants were adults with subacute and chronic stroke who had been admitted for rehabilitation to two hospitals in Madrid, Spain (Hospital Los Madroños and Hospital Universitario 12 de Octubre). 

Inclusion criteria were age between 30 and 80 years, presenting hemiparesis or hemiplegia of the left upper limb, right hand dominance, presenting sensory alterations in the affected limb as a consequence of the lesion after neurological assessment, and at least six months since the onset of the stroke. Those with aphasia, apraxia, concomitant pathologies affecting mobility or sensibility, absence of limitation of mobility, sensory deficits in the affected limb, or cognitive deficits expressed as a score of less than 24 points on the Mini-Mental State Examination [26] were excluded.

Participants were recruited following a consecutive non-probability sampling and were assigned to one of two groups (control or experimental) according to their geographical distribution. The control group (n = 9) received conventional rehabilitation treatment, while the experimental group (n = 9) received a robotic intervention with intensive high-frequency vibration system Amadeo^®^ in addition to conventional treatment (Tyromotion GmbH, Graz, Austria, 2016). The Amadeo system© version 5.1. is an end-effector robotic system that provides a vibratory proprioceptive stimulus of different frequencies and intensities through a support arm and equipment that is strapped to the forearm and wrist of the seated patient. Each finger is equipped with a small, magnetized plate at the fingertips. Following published stimulation recommendations, vibrations were provided at a maximum frequency of 60 Hz for 20 min per session. This intervention has been previously described in further detail [21].

The study was conducted between September 2020 and August 2021. The variables of interest were collected through an anonymized and individualized dossier that included the socio-demographic and medical data of the participants, and the results of the different measurements used. These measurements were applied by evaluators external to the research group and blinded to the intervention. These evaluators had been previously trained in the use and interpretation of the tools. All measurements specific to the research were collected in the same order for all participants at a time that did not coincide with their usual treatment. Measurements included Semmes-Weinstein Monofilaments^®^ (North Coast Medical, Inc., Morgan Hill, CA, USA, 2011), Fugl-Meyer Assessment Upper Extremity Scale, Motor Activity Log, and Stroke Impact Scale.

### 2.3. Measurements

The following instruments were administered before and after intervention: Semmes-Weinstein Monofilaments^®^ (to measure sensory function), the Fugl-Meyer Assessment Upper Extremity Scale (to measure motor and sensory function), the Motor Activity Log (to assess upper extremity motor performance), and the Stroke Impact Scale (to assess global performance in basic and instrumental activities of daily living).

Semmes-Weinstein Monofilaments^®^ are the most widely used and accurate instrument for measuring sensory function in people with stroke [27]. In the present study, we used the Touch-Test hand kit, which consists of five monofilaments of nylon fibers of different thicknesses, where a thickness greater than 2.83 indicates worse sensory function [28]. The monofilaments were applied to all areas corresponding to the dermatomes on the anterior and posterior sides of the affected side, which were further classified into hand, forearm, arm, and shoulder [29,30,31].

The Fugl-Meyer Assessment is one of the most commonly used tools to measure motor and sensory function in people with stroke [32]. It consists of five domains, including motor function, which can be administered independently. For this study, the upper extremity domain was used [33]. Each item is scored on a three-point scale (0 = cannot be performed; 1 = partially performed; 2 = fully performed), with a total score of 66 points, where a higher score indicates a greater deficit. In addition, it provides three other subscales measuring sensation, passive joint movement, and joint pain of the upper limb.

The Motor Activity Log is a tool that assesses the quantity and quality of upper limb movement in the performance of daily activities [34]. Each item is scored on a six-point scale that accepts partial scores, where a higher score indicates a better performance. This tool provides two total scores, one for the quantity of movement subscale and another one for the quality of movement subscale [35].

Finally, the Stroke Impact Scale assesses performance on different basic and instrumental activities of daily living in people with brain injury. Each item is scored on a five-point scale, where a higher score indicates a better performance [36]. We used the 16-item version, which has been widely used in previous research [37].

### 2.4. Statistical Analyses

The statistical analyses were performed using SPSS (IBM SPSS Statistics version 27.0 for Windows, IBM Corp., Armonk, NY, USA, 2022). Categorical variables were expressed in absolute and relative values, and the mean, standard deviation, median, and interquartile range of numerical variables were calculated according to their distribution. Since the outcome variables did not fit a normal distribution (Shapiro–Wilk test *p* < 0.05), non-parametric bivariate analyses were used. First, both groups were tested for differences in function and performance variables using the Mann–Whitney U-test. The association between pre-intervention function and performance variables of both groups was explored using Spearman correlation coefficients. The variables that were significantly associated with the performance measures (MAL and SIS-16) in the bivariate analyses were included in the linear regression models, considering the post-intervention performance variable of the experimental group as the dependent variable and the pre-intervention measurement and significantly correlated measures as independent variables.

## 3. Results

A total of eighteen participants completed the intervention and thus were included in the analyses. No adverse effects were reported for any group. The socio-demographic and clinical characteristics of both groups can be found in Table 1.

Both groups had similar levels of sensory and motor function and upper limb and global performance prior to the intervention, except for the baseline upper limb quantity of movement subscale, which was better for the experimental group (Table 2).

As shown in Table 3, small to large statistically significant correlations were found between the different variables, but these associations were not uniformly present. Sensory function of the different areas of the upper limb was found to be closely correlated in all areas (r = 0.772–0.971, *p* < 0.05). However, sensory function measured with the Semmes-Weinstein Monofilaments^®^ was not associated with upper limb motor or sensory function as assessed with the FMA, nor with any measure of global or upper limb performance. Motor function was associated with upper extremity performance and daily performance of basic and instrumental activities of daily living and was therefore included as an independent variable in the following multivariate regression models (r = 0.513–0.582; *p* < 0.005). In addition, passive joint mobility was included as an independent variable for the performance of quality and quantity of upper limb movement (r = 0.523–0.547; *p* < 0.005), and upper limb pain was included as an independent variable for the overall performance of basic and instrumental activities of daily living (r = 0.461; *p* = 0.054).

Regarding the results of the multivariate analysis shown in Table 4, the 97.6–99.0% variance in post-intervention upper extremity movement quantity and quality performance was explained by previous movement quantity and quality and previous motor function and passive joint movement (F = 81.747–195.294; *p* < 0.001). However, only prior motor function individually contributed significantly to movement quantity, although all independent variables (prior quantity and quality of movement and prior motor function and passive joint movement) contributed significantly to post-intervention movement quality.

Regarding post-intervention performance in basic and instrumental activities of daily living, although the proposed model as a whole explained 78.8% of the variance (F = 10.927; *p* = 0.012), it was only significantly explained by the pre-intervention level of daily performance.

## 4. Discussion

The aim of this study was to examine the relationship between upper limb motor and sensory functions, performance in upper limb activities in terms of quality and quantity of movement, and performance in basic and instrumental activities of daily living in people with subacute and chronic stroke before and after robot-assisted intervention.

Our findings show that sensory function measured with Semmes-Weinstein Monofilaments^®^ was not related to upper limb activity performance assessed with the MAL, nor to basic and instrumental activity performance assessed with the SIS-16. In contrast, motor function and passive upper limb joint movement were independently associated with upper limb movement quality and quantity performance (r = 0.523–0.583; *p* < 0.05), and motor function was also independently and significantly correlated with daily global performance (r = 0.513; *p* < 0.05). However, in multivariate regression models, the influence of these factors differed for each aspect of performance. For instance, motor function significantly contributed to the quality and quantity of movement, but not to the performance of basic and instrumental activities of daily living once the pre-intervention overall performance level was controlled for.

These results are partially consistent with those reported in the previous scientific literature. Different studies, both cross-sectional and longitudinal, have shown that upper limb body functions contribute to explaining different aspects of upper limb capacity and performance in people with stroke. Harris and Eng [37] reported that the strength of the paretic upper limb explains between 78% and 87% of the variance of arm activity in this population. Regarding sensorimotor function, a recent study showed that upper limb sensorimotor function directly influences the quality and quantity of movement in upper limb activities and, in turn, indirectly influences the daily performance of basic and instrumental activities of daily living through both factors [38]. In addition, findings from Keeling et al. [25] showed that both motor function and upper limb and overall performance improved following a robot-enhanced intervention in combination with conventional rehabilitation. However, this study did not specifically evaluate whether function influenced performance recovery. In this sense, our study further expands on these findings, as motor function and passive joint mobility only had a direct effect on the quality and quantity of upper limb movement, but not on overall performance. On the other hand, in our sample, pain showed a moderate but non-significant correlation with upper limb activity performance. Previous studies reported that the baseline intensity of pain in the paretic shoulder is related to motor performance, quality of life, and motor functions, and negatively impacts the recovery of upper limb function [39]. It is possible that the absence of a significant correlation in our work is partly explained by a lack of statistical power as a result of the small sample size.

The relevance of deficits in body functions and structures on people’s activities and participation is a topic of great interest in the scientific literature. For instance, improvements in body functions do not always lead to improvements in performance, and the circumstances under which this does occur are not clear [24,40]. In this regard, a recent systematic review concludes that deficits in motor, emotional, executive, and cognitive functions, in combination with activity limitations, significantly predict the social participation of people with stroke in their community [41]. Moreover, there is evidence that performance and ability in daily activities are able to predict post-stroke participation more accurately than deficits in body functions [7,42], with the exception of executive, psychological, and cognitive functions [7,42,43]. However, there is no consensus on this aspect, as other studies indicate that motor function, in combination with executive and psychological functions, different personal factors, and the performance of basic activities of daily living, predict the performance of other more complex tasks in the stroke population, such as instrumental activities of daily living [14]. In addition, sensorimotor function of the upper limb, and especially of the lower limb, including strength and coordination, also directly predict participation restrictions in people with stroke [42,44]. This could help explain why, in our sample, the overall performance of basic and instrumental activities of daily living was mostly explained by previous general performance, as we only assessed the sensorimotor function of the upper limb, and most daily activities require the involvement of global body function.

Overall, it is possible that the effect of upper limb sensorimotor functions on global performance and participation is partially mediated by upper limb movement quality and quantity performance, as proposed by Hiraga et al. [38], and that lower limb motor function and performance contribute significantly to this mediating effect [42]. However, it is important to highlight that none of the studies discussed here evaluated this effect after robotic intervention, so our results provide new information regarding how this relationship contributes to improving function after intervention.

Given the important impact that previous motor function and performance seem to have on the improvement of global and upper limb performance post-intervention, it is necessary to investigate the effect of the different intervention strategies available for the stroke population on these specific variables. In this respect, the scientific literature is inconclusive. One clinical trial concluded that although a robotic-assisted therapy improved deficits in sensorimotor functions of the upper limb, this improvement did not translate into increased performance in activities or improved quality of life [45]. In this regard, a recent systematic review and meta-analysis concluded that this type of intervention can improve not only sensorimotor function but also activity performance in the stroke population, but only if applied for a total of 15 h or more and in patients with a high level of participation [46]. The challenge of how to translate improvements in body functions to activity performance and participation is common to other intervention approaches in this population, such as nerve stimulation [47]. The existence of promising preliminary results in this regard, in relation to augmented reality and mixed reality therapy [48], may provide an interesting starting point for testing the efficacy of a therapy combining this type of intervention with a robotics-supported approach on activities and participation. Therefore, our findings provide new information regarding how baseline levels of sensorimotor function and upper limb and overall daily performance may influence recovery success using a combination of conventional occupational therapy intervention and robotic intervention.

### Limitations

The present research has several limitations that need to be addressed. First, the sample size is not large enough to allow us to extrapolate our results to stroke patients under other conditions considered in our inclusion criteria and other neurological diseases. However, its statistical power may be insufficient to detect moderate significant correlations or to perform a more in-depth type of analysis, such as structural equation modelling, which would have allowed us to examine the mediating role of upper limb performance in the relationship between upper limb sensorimotor function and performance in basic and instrumental activities of daily living. Furthermore, the sample was heterogeneous in different socio-demographic variables, which limits its representativeness. Finally, the absence of a control group to compare our findings with and of a follow-up assessment and an evaluation of lower limb sensorimotor function should be corrected in future studies. Nevertheless, the findings of this study provide relevant information on the influence of previous motor function and performance on the improvement of upper limb and global performance in stroke patients after an intervention supported by a high-frequency vibration robotic system.

## 5. Conclusions

The results of this study suggest that basal upper limb motor function is significantly associated with improved performance in terms of the quantity and quality of movement following a combined intervention of conventional treatment and a robotic vibration stimulation system in people with stroke. These findings should be further explored in future studies involving larger samples and follow-ups.

## Figures and Tables

**Table 1:** Sociodemographics of control and experimental groups (n = 18).

	Control Group (n = 9)	Experimental Group (n = 9)	*p*-Value
Age (M [SD])	72.89 (10.20)	66.56 (9.88)	0.200
Range of age (years)	54–85	45–77	-
Sex			0.046 *
Male [N (%)]	4 (44%)	8 (89%)	
Female [N (%)]	5 (56%)	1 (11%)	
Disease duration in months (M (SD))	8.44 (4.64)	9.22 (3.38)	0.690
Range of disease duration (months)	3–14	4–12	-

Note: FMA-UE = Fugl-Meyer assessment of the upper extremity; MAL = Motor Activity Log; SIS-16 = Stroke Impact Scale-16. * *p* < 0.05.

**Table 2:** Baseline function and performance variables of control and experimental groups (n = 18).

Variable		Median (Interquartile Range)		*p*-Value
		Control Group (n = 9)	Experimental Group (n = 9)	
Upper extremity sensation (Semmes-Weinstein Monofilaments^®^)	Hand	4.31 (5.65)	3.61 (2.02)	0.858
	Forearm	4.31 (5.65)	4.31 (3.80)	0.929
	Arm	3.61 (6.65)	4.31 (3.85)	0.718
	Shoulder	3.81 (6.15)	4.31 (2.80)	0.787
Upper extremity motor and sensory impairment (FMA-UE)	Motor Function	33.00 (47.50)	14.00 (45.00)	0.965
	Sensation	4.00 (9.00)	6.00 (2.50)	0.622
	Passive Joint Motion	20.00 (7.50)	20.00 (1.50)	0.822
	Joint Pain	20.00 (7.00)	20.00 (6.50)	0.436
Upper extremity performance (MAL)	Amount Scale	0.00 (22.00)	15.00 (25.50)	0.048 *
	How Well Scale	0.00 (21.00)	14.00 (15.50)	0.079
ADL and IADL performance (SIS-16)	Daily Impact	53.00 (34.50)	46.00 (19.00)	0.536

Note: FMA-UE = Fugl-Meyer assessment of the upper extremity; MAL = Motor Activity Log; SIS-16 = Stroke Impact Scale-16. * *p* < 0.05.

**Table 3:** Correlations between function and performance variables at baseline (overall group, n = 18).

	1	2	3	4	5	6	7	8	9	10
1. Sensation Hand	**-**									
2. Sensation Forearm	**0.809**	-	-							
3. Sensation Arm	**0.772**	**0.888**	**-**							
4. Sensation Shoulder	**0.774**	**0.808**	**0.971**	-						
5. FMA-UE Motor Function	0.009	0.086	0.006	0.017	-					
6. FMA-UE Sensation	0.317	0.203	0.335	0.344	**0.519**	-				
7. FMA-UE PJM	0.072	0.018	0.071	0.088	**0.591**	**0.710**	-			
8. FMA-UE Pain	0.001	0.153	0.137	0.050	0.466 ^a^	**0.479**	**0.733**	-		
9. MAL Amount	0.151	0.202	0.128	0.135	**0.567**	0.445	**0.523**	0.370	-	
10. MAL How Well	0.164	0.240	0.124	0.112	**0.582**	0.454	**0.547**	0.354	**0.991**	-
11. SIS-16	0.170	0.293	0.233	0.211	**0.513**	0.438	0.414	0.461 ^b^	0.274	0.324

Notes: FMA-UE = Fugl-Meyer assessment of the upper extremity; PJM = passive joint mobility; MAL = Motor Activity Log; SIS-16 = Stroke Impact Scale-16; in bold = *p* < 0.05; ^a^ *p* = 0.051; ^b^ *p* = 0.054.

**Table 4:** Multivariable regression model of the performance measure post-intervention scores in the experimental group.

	**MAL Amount Subscale** **(Post-Intervention)**		
	**β (SE)**	***t***	***p*-Value**
MAL Amount Subscale (pre-intervention)	−0.59 (0.29)	−2.049	0.110
FMA-UE Motor Function (pre-intervention)	0.85 (0.11)	7.397	**0.002**
FMA-UE PJM (pre-intervention)	1.61 (0.68)	2.376	0.076
MAL How Well Subscale (pre-intervention)	0.83 (0.43)	1.924	0.127
Adjusted R2 (%)	97.6%		
Model	F = 81.747; ***p* < 0.001**		
	**MAL How Well Subscale** **(post-intervention)**		
	**β (SE)**	***t***	***p*-value**
MAL How Well Subscale (pre-intervention)	1.43 (0.26)	5.412	**0.006**
FMA-UE Motor function (pre-intervention)	0.89 (0.07)	12.705	**<0.001**
FMA-UE PJM (pre-intervention)	1.59 (0.41)	3.830	**0.019**
MAL Amount Subscale (pre-intervention)	−1.27 (0.18)	−7.139	**0.002**
Adjusted R2 (%)	99.0%		
Model	F = 195.294; ***p* < 0.001**		
	**SIS-16 ADL and IADL Impact** **(post-intervention)**		
	**β (SE)**	***t***	***p*-value**
SIS-16 ADL and IADL Impact (pre-intervention)	0.82 (0.19)	4.384	**0.007**
FMA-UE Motor Function (pre-intervention)	0.11 (0.10)	1.097	0.323
FMA-UE Pain (pre-intervention)	−0.20 (0.38)	−0.520	0.625
Adjusted R2 (%)	78.8%		
Model	F = 10.927; ***p* = 0.012**		

Notes: SE = standard error; FMA-UE = Fugl-Meyer assessment of the upper extremity; PJM = passive joint mobility; MAL = Motor Activity Log; SIS-16 = Stroke Impact Scale-16; in bold = *p* < 0.05.

## Data Availability

Data is not available due to privacy and ethical restrictions.
